# Sustainable biofuel synthesis from non-edible oils: a mesoporous ZSM-5/Ni/Pt catalyst approach

**DOI:** 10.1039/d4ra00346b

**Published:** 2024-03-05

**Authors:** V. L. Mangesh, Tamizhdurai Perumal, S. Santhosh, Nadavala Siva Kumar, A. Vijayaraj, G. S. V. Seshu Kumar, S. Sugumaran, G. Murali, Praveen Kumar Basivi, Ahmed S. Al-Fatesh

**Affiliations:** a Department of Mechanical Engineering, Koneru Lakshmaiah Education Foundation Vaddeswaram Guntur Andhra Pradesh 522502 India vlmangesh@gmail.com; b Department of Chemistry, Dwaraka Doss Goverdhan Doss Vaishnav College (Autonomous) (Affiliated to the University of Madras, Chennai) 833, Gokul Bagh, E. V. R. Periyar Road, Arumbakkam Chennai 600 106 Tamil Nadu India tamizhvkt2010@gmail.com +91 9677146579; c Sagi Rama Krishnam Raju Engineering College Bhimavaram Andhra Pradesh 534204 India; d Vishnu Institute of Technology Bhimavaram Andhra Pradesh 534202 India; e Department Chemical Engineering, College of Engineering, King Saud University P. O. Box 800 Riyadh 11421 Saudi Arabia; f Pukyong National University Industry-University Cooperation Foundation, Pukyong National University Busan 48513 Republic of Korea

## Abstract

This work examines the hydrodeoxygenation (HDO) activity of non-edible oils using a high surface area catalyst. The HDO activity was thoroughly examined and contrasted using the high surface area catalyst Ni/Pt-ZSM-5 as well as other supports like MCM-48 and H-beta. Ni/Pt bimetals supported on mesoporous ZSM-5 were created *via* reverse order impregnation to facilitate HDO of non-edible oils. Techniques such as XRD, FT-IR, BET, HR-TEM, HR-SEM, TPD, and TGA were used to characterize the produced catalysts. The synthesized catalysts considerably influenced the hydrodeoxygenation activities for the synthesis of lengthy chain hydrocarbons in a stainless-steel reactor with a high-pressure fixed bed between 300 and 375 °C under 10–40 bar hydrogen pressure. High levels of Ni/Pt-ZSM-5 acidity, textural, and H_2_ consumption qualities were discovered. Distributions of the products were also reviewed, along with comparisons of the structure–activity connections.

## Abstract 2

This work examines the hydrodeoxygenation (HDO) activity of non-edible oils using a high surface area catalyst.

## Graphical abstract

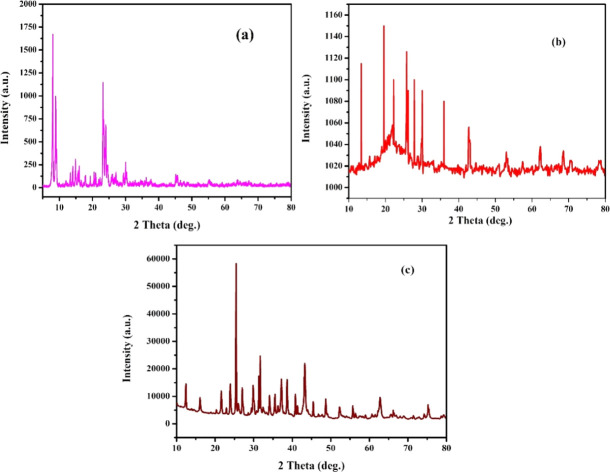

## 1. Introduction

Global biofuel research is necessary because future transportation will need to be less dependent on fossil fuels and emit fewer pollutants. It is more environmentally friendly, sustainable, and renewable to produce biofuel from biomass. The procedures' efficiency and environmental friendliness reduce the need for fossil fuels. Numerous, excellent studies have been conducted with the goal of producing ultra-clean biofuel energy through several catalytic hydrotreatment techniques.^1–3^ Particular focus has been placed on hydrodeoxygenation (HDO), which uses lignocellulosic biomass to produce sustainable, renewable biofuels and chemicals.^4^ The initial extracts of oil from bio sources have disadvantages such as high oxygen content, which initiates the propensity to polymerize during storage and transportation, and being corrosive and immiscible with fossil fuels. Once more, they are caustic, have a low heating value, and are highly viscous.^5^ This calls for upgrading the oil extracts to meet gasoline specifications. There is a great need for renewable hydrocarbon fuels on a global scale, and rules to lessen the negative effects of industry on the environment are also being implemented.^6–8^ Both the industrial need for an alternative fuel that is free of pollutants and the need for HDO will be satisfied by the HDO reaction.^9,10^ Previous studies have shown the transformation of non-food oils such as long chain fatty acids, palm oil, jatropha oil, and microalgae oil into hydrocarbons using a variety of catalysts.^11,12^ Mesoporous materials, such as zeolites and silica derivatives, have garnered significant attention across various fields due to their unique properties.^13^ These materials possess high surface area, large pore volume, and tunable pore size distribution, making them highly desirable. Moreover, mesoporous materials have proven to be effective in environmental remediation. Noble metals on zeolite supports are frequently utilized, and their yield in HDO is significantly higher than that of conventional catalysts. However, larger application in industry is limited by the increased cost.^14^ In the energy sector, mesoporous materials such as zeolites and silica derivatives have great potential in several applications.^15–20^ For instance, zeolites have been utilized as catalysts for crucial energy-related processes such as hydrocarbon cracking and isomerization. The zeolite materials' shape selectivity significantly affects the guaiacol HDO.^21^

Noble metals including Pd, Ru, Pt, and Pt–Re were also used in the hydro-treatment of bio-oils to create synthetic fuel, and excellent activity has been documented.^22–25^ Noble metal catalysts need high-pressure reactions; nevertheless, their high cost and scarcity of deposits prevent them from being used on a broader scale.^26,27^ Therefore, efforts have been focused on attempting to use non-noble metals as catalysts with the same efficacy. Transition metals like Ni, Co, Mo have been discovered to be effective HDO reaction catalysts and promoters. HDO of microalgae oil, jatropha oil, palm oil, and long chain fatty acids for the generation of hydrocarbons and fuels was determined to be Ni(Co)/Mo(W) supported on commercial-Al_2_O_3_ catalysts.^28,29^ Researchers explored the selective hydrogenation and deoxygenation of vegetable oil using a Ni–Mo catalyst to construct a realistic reaction mechanism.^30^

MoO_3_–NiO catalysts supported on mixed metal oxides such as TiO_2_, ZrO_2_, CeO_2_, and Al_2_O_3_ were proven to exhibit activity in the hydrodeoxygenation of lignin model compounds, including esters, phenolic compounds, cyclic ketones, and aldehydes. These reactions were carried out under reduced pressure and hydrogen flow.^31,32^ Supports made of TiO_2_, ZrO_2_, CeO_2_, and Al_2_O_3_ have certain limitations such as reduced surface area, ineffective active metal spice dispersion, and leaching. Unique catalytic capabilities which include reduced deactivation, bifunctional zeolite-supported transition metal catalysts received more attention.^33^ For instance, because of their activity in a variety of catalytic processes and in HDO, Ni-based catalysts have garnered a lot of attention.^34^ The average size of the active component's particles is an important reaction parameter, and increasing the metal dispersion improves both the catalytic efficiency and the catalyst lifespan. All these factors favour zeolites for HDO and this study utilized Ni–Pt bimetal catalysts on zeolites for the HDO of jatropha oil.^35,36^ Again, noble metals like Pt are costly but they do provide higher efficiency in conversion with extended catalyst life. The non-sulfide mixed metal catalyst under neutral and acidic support is the subject of the current study. This catalyst is crucial for the conversion and selectivity of HDO of jatropha oil.^37^ Natural zeolites are cheap and widely available, and using them in conjunction with Ni has produced positive outcomes for anisole HDO. Crude bio-oil products that are not edible are a highly favoured fuel for the hydrodeoxygenation process because they yield hydrocarbons. These hydrocarbons are as energetically dense as common fossil fuels such as diesel, jet fuel, light naphtha and kerosene. Mesoporous substances with pores 2 nm to 50 nm in size, such as KIT-6, HMS, MCM-48, SBA-16, SBA-15, and MCM-41, have been used as supports in recent years to disseminate the active metal species in HDO reactions.^38,39^

In the present study, we conducted a comprehensive investigation and comparison of the hydrodeoxygenation (HDO) activity using a high surface area catalyst Ni/Pt-ZSM-5, for the conversion of non-edible oils. Investigated and compared the HDO activity in detail utilizing the high surface area catalyst Ni/Pt-ZSM-5, and other supports including MCM-48, and H-beta. The tests were performed in a high-pressure fixed-bed reactor made up of stainless-steel at pressurized conditions ranging from 10 to 30 bar and at different temperatures. The catalysts' structural activity connection has been clarified. It was discovered that the HDO activity is influenced by the varied acid functionalities and textural characteristics of mesoporous ZSM-5 supports. The support ZSM-5 used in this study was made using a straightforward sol–gel process and has excellent surface shape and textural qualities.

## 2. Experimental methods

### 2.1. Synthesis of ZSM-5 zeolite

The procedures described below are an altered version of previously described processes.^40^ C_26_H_58_ClNO_3_Si [3-(trimethoxysilylpropyl) octadecyl dimethyl ammonium chloride], 75% solution of CH_3_OH and (CH_3_CH_2_CH_2_)_4_N(Br) (TPABr) were utilized as pattern to develop ZSM-5 zeolite molecular sieves from a reaction gel. The following was done to prepare a conventional synthesis gel: in order to make solution A, 52.4 g of distilled water was mixed vigorously for 30 minutes at the temperature of the room with 0.6 g of NaOH pellets, 0.7 g of metakaolin and 1.8 g of tetrapropyl ammonium bromide (TPABr), until the mixture was homogenous. After continued stirring for a period of two hours, a homogeneous mixture consisting of 4.346 g of Si(OC_2_H_5_)_4_ (TEOS) and 0.654 g of octadecyldimethylammoniumchloride (ODAC) was added to the final mixture of A to create a gel. The mixture was then put into an autoclave made of brushed stainless steel and heated for 24 hours at 120 °C. The product was thoroughly washed, dried out overnight at 100 °C, and then calcined in presence of air at 550 °C for a period of six hours at a rate of heating 1 °C per minute.

### 2.2. Preparation of MCM-48 zeolite

First take 4.8 g of cetrimonium bromide (CTAB) in 45 mL deionized water. After adding 0.6 g of aluminium nitrate, the mixture was rapidly agitated for 45 minutes. The mixture was then given 60 mL of HP-grade ethanol and 10 mL of 34 weight percent ammonia, and it was continuously agitated for 30 minutes. The mixture was then given a second addition of 7.2 g of TEOS (tetra ethyl *ortho* silicate), and it was vigorously agitated for 48 hours. For 48 hours, the premixes were continually mixed at room temperature. The collected mixture was filtered after being air dried for 48 h at 100 °C. The sample was ultimately heated to 550 °C for 5 hours.^41^

### 2.3. Preparation of H-beta zeolite

0.867 g of aluminium sulphate (Al_2_(SO_4_)_3_·18H_2_O), 0.65 g of sodium hydroxide (NaOH), and 6 g of 40% aqueous TEAOH are combined and stirred till translucent after 45 minutes. The mixture was then agitated for another hour or so while 3.2 g of silica was added. This produced a thick gel that was then dried completely at 60 °C. The resulting dry precursor pieces were then roughly smashed and placed in a 4 mL of Teflon cup, which was then placed inside a 20 mL Teflon liner. The 0.2–0.543 mL of water was put into the liner bottom without contacting the dry gel in the internal Teflon cup. The electrified liner assembly was placed in a 20 mL reactor made up of steel. The oven was prepared to 150 °C, heated for between 2 and 3 days, and then the reaction that occurred was quenched. The transformed gel was filtered cleaned, then dried. The template was removed by calcining in air at a temperature gradient of 2 °C per min to 550 °C for 6 hours. Elemental analysis of the calcined sample yielded Si/Al ratios near to the theoretic values mentioned in the text. Solutions containing particles of colloidal nature were filtered three times in centrifuged water at twenty thousand rpm for a minimum of 45 minutes. The sample was ultimately heated to 550 °C for 5 hours.^42^

### 2.4. Ni metals impregnation on supports

The catalysts (ZSM-5, MCM-48, H-beta) in this study were loaded with nickel (1%), which was done by impregnating them with an aqueous solution of Ni(NO_3_)_2_ (nickel(ii) nitrate hexahydrate), drying them at 120 °C for a period of six hours, and then calcining them in an air flow at 550 °C for two hours.

### 2.5. Platinum metals impregnation on supports

To impregnate platinum (0.5%) on the catalysts, they had been added with H_2_PtCl_6_ aqueous solution, dried at 120 °C for a duration of six hours, and calcined at 450 °C in an air flow for two hours. The reduced state of platinum was maintained at hydrogen flow at 200 °C prior to the catalyst being transferred into the reactor for HDO.

### 2.6. Catalytic activities

The HDO activity of the catalyst has been determined using a vapour phase fixed bed high-pressure reactor. In this study, jatropha oil that had been dissolved in decalin and was put to use as a liquid feed and high purity hydrogen were used as feed. The display screen kept an eye on the temperature and pressure. 3.2 g of catalyst particles (15 mm in diameter and 380 mm in length) were positioned inside the bed, along with glass beads and inert silica wools as support. Before reaching the catalyst bed, the liquid feed undergoes the vaporization process for proper mixing with catalyst. The HDO of jatropha oil was performed by varying factors like temperature (300 to 375 °C), pressure (30 bar), and WHSV. Both the liquid and the gas were collected after the reaction was finished. The product was collected at various periods and analyzed using the gas chromatograph (Shimadzu-GC-17A with DB-5 column). The jatropha oil conversion was calculated using both the final and initial concentrations (mole) acquired from GC.

### 2.7. Catalyst characterization

The several physical–chemical methods were used to characterize all of the synthesized catalysts, including low and high angle XRD. N_2_ physisorption investigations, temperature programme desorption (TPD), and FTIR. The BRUCKER D8 diffractometer was used to capture the diffraction pattern of the supports and catalysts at low angles of 0.5–5° and high angles of 10–80°, with a step scan rate of 0.02 seconds using Cu K-radiation (*λ* = 1.548 Å). The N_2_ sorption isotherms were measured at liquid N_2_ temperature using the QUADRASORB SI automated analyzer. Before the analysis, 0.03 g of the sample was degassed at 300 °C for 4 hours with N_2_ flow. The specific surface area values of the supports and catalysts were computed using the BET technique. The pore volume (*V*_p_) has been determined using nitrogen adsorption at a relative pressure range of 0.98, and a graph of the distributions of pore sizes was generated from the desorption isotherms using the BJH method. Temperature-programmed desorption tests were performed on the Micrometrics chemisorb 2750 TPD utilizing gas combinations of 5% H_2_/95% Ar and 10% NH_3_/90% He, respectively. Prior to analysis, 30 mg of sample was degassed at 200 °C for 3 hours with N_2_ gas flow. The Fourier transform infrared (FT-IR) spectrum was acquired with a PerkinElmer (FT-IR C101375) Spectrophotometer. The samples were prepared using the KBr pellet technique. The HR-TEM images of the samples were taken with a JEOL equipment (JEM 2010). The size and morphology of catalysts were investigated using a scanning electron microscope (SEM) (a Zeiss paired) operating at 3 kV. 3–5 mg of catalyst was dissolved in 2 mL of methanol and then sonicated for 20 minutes.

## 3. Results and discussion

### 3.1. X-ray diffraction analysis

**Fig. 1:**
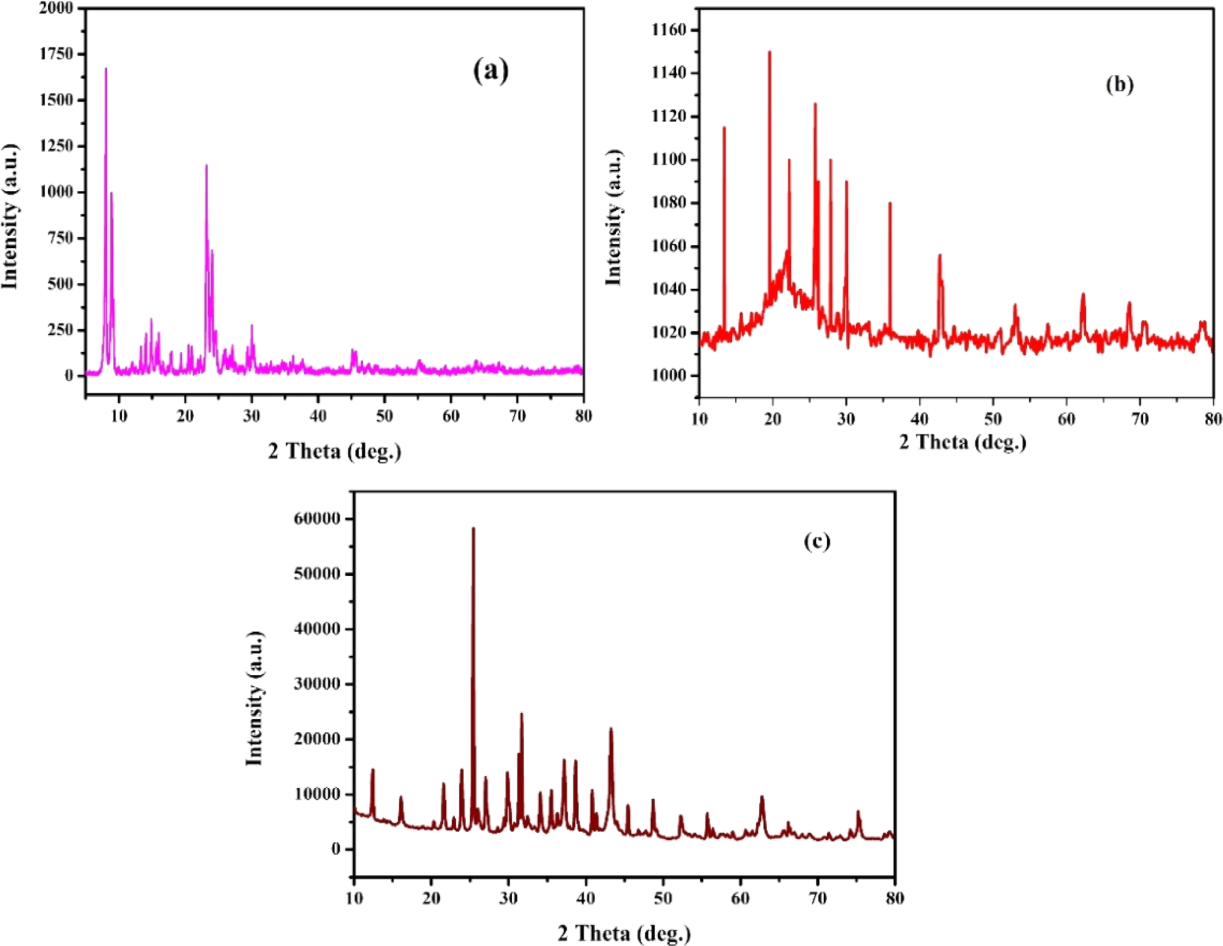
High angle XRD pattern of (a) Ni/Pt-ZSM-5, (b) Ni/Pt-MCM-48, and (c) Ni/Pt-H-beta catalyst.

Fig. 1 shows the X-ray diffraction pattern of ZSM-5 (JCPDS 00-44-0003), MCM-48 (JCPDS 00-049-171), and H-beta (JCPDS 48-0038) zeolites with different Si/Al ratios. The XRD patterns of the synthesized catalysts were dominated by the characteristic peaks of the zeolite framework. MCM-48 has been identified as mesoporous silica with cubic porous structure by detecting peaks at (211) and (220) which characterize the *Ia*3*d* space group^41,43^ (JCPDS 00-049-171) seen in (Fig. 1b). Ni/Pt-H-beta catalysts exhibit wide reflections at 2*θ* = 22.5°, 25.4°, 27.0°, 28.8°, and 29.7°, which are associated with the beta zeolite according to (JCPDS 48-0038) data. On the other hand, the catalysts supported on ZSM-5 zeolite (Fig. 1a) show reflections at 2*θ* = 13.9°, 15.9°, 23.1°, 24.0°, 25.9° and 27°, which are attributed to the MFI structure type. In general, all the calcined catalysts exhibit peaks that correspond well with the Bragg reflections of the standard face-centred cubic (fcc) structure, with peaks at 2*θ* = 43.2°, 50.8°, and 73.2° being assigned to their characteristic (111), (200), and (220) indices. These indices correspond to the reflections of NiO (JCPD no. 04-0835).^21^ After depositing Pt on Ni nanoparticles *via* a reduction reaction, all the catalysts Ni/Pt-ZSM-5, Ni/Pt/MCM-48, and Ni/Pt-H-beta show diffraction peaks at 39.8°, 45.8°, and 67.4°, which can be assigned to (111), (200), and (220) planes of the metallic Pt nanoparticles (JCPDS card no. 04-0802).^44^ This suggests that the structure of Pt material layers was not affected by the structural difference of Ni nanoparticles. The well-separated diffraction peaks in (Fig. 1) suggest the presence of different phases indicating that no alloy phase was formed. Furthermore, diffraction peaks at 52.4° and 60.7° match with a metallic Ni (JCPD no. 04-0850) structure assigned to the Ni-core.^45^ The Ni/Pt-ZSM-5, Ni/Pt/MCM-48, and Ni/Pt-H-beta samples all exhibit a well-defined crystalline structure, evidenced by the presence of sharp peaks. However, since the macrospore pore walls were not completely converted into MFI framework, the highest achievable intensity of the Pt-ZSM-5 zeolites decreased as the Si/Al ratio increased. The average size of the crystal particles in the synthesized Ni/Pt-ZSM-5, Ni/Pt/MCM-48, and Ni/Pt-H-beta zeolites was determined using the Debye–Scherrer formula. This formula calculates the mean crystallite size, *L*, using the wavelength of X-rays, *λ*, the angle of the Bragg diffraction pattern, *θ*, and the full width at half maximum (FWHM) of the source's peak, *β*. To determine the peak position and width accurately, two Gaussian curves were fitted to the peaks using monochromatic CuKα radiation. According to the Scherrer formula, the average crystallite diameters for the Ni/Pt-ZSM-5, Ni/Pt/MCM-48, and Ni/Pt-H-beta zeolites were 35.1, 36.9, and 37.6 nm, respectively.^46–48^

### 3.2. FT-IR analysis

**Fig. 2:**
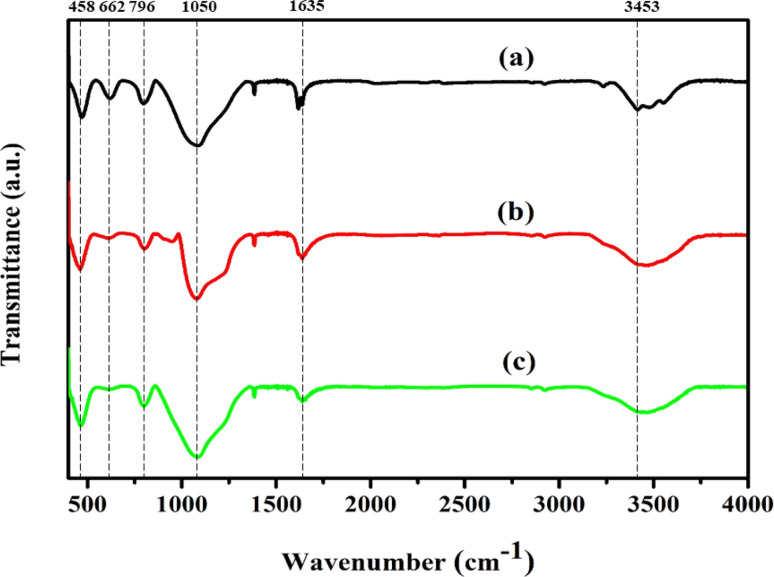
FT-IR spectrum of (a) Ni/Pt-ZSM-5, (b) Ni/Pt-MCM-48, and (c) Ni/Pt–H-beta catalyst.

FT-IR analysis was performed to obtain additional evidence for the development of each zeolite crystal phase. In zeolite chemistry, infrared spectroscopy has been used to provide information on zeolite synthesis, adsorption, framework vibrations, surface characteristics, and catalysis.^49^Fig. 2 shows the FT-IR spectra used to describe the silanol groups, as well as the stretching and bending vibrations of Ni/Pt-ZSM-5, Ni/Pt/MCM-48, and Ni/Pt-H-beta. Absorption bands are visible at wavelengths of 3640, 3453, 1635, 1220, 1150–1050, 796, 553 and 458 cm^−1^. The spectra of all the samples are identical in terms of band locations.^50^ The absorption wavelengths at 458 cm^−1^, 1796 cm^−1^, 1100 cm^−1^, and 1220 cm^−1^ correspond to the T–O bending, interior asymmetric stretching, external symmetric stretching, and exterior asymmetric stretching vibrations of the siliceous materials. The bands at 1200 to 450 cm^−1^ can be assigned to Si–O–Al, Si–O–Si, Si–O, Si–Al and T–O species. On the other hand, the bands observed between 3750 to 3450 cm^−1^ are attributed to Si–OH, Si–OH–Al, and –OH hydroxyl groups. The absorption band with high intensity around 3714 cm^−1^ is assigned to internal silanol groups.^33^ After ion-exchange and recalcination under vacuum in infrared cell, the intensity of this peak decreased, which was also observed by others. This decrease is explained as the facile annealing of the hydrogen-bonded silanol groups generated on aluminium extraction. Moreover, the band present at 3601 cm^−1^ represents OH groups bonded with extra-framework aluminium species (Al–OH). Similarly, the absorption band around 3640 cm^−1^ is ascribed to the O–H stretching vibration of Brønsted acid OH groups from the bridging Si(OH)Al in the zeolite framework.^51,52^ Lastly, the band at 662 cm^−1^ is assignable to Si–O–M where M is the exchangeable Ni^+^ ion metal species. All three types of zeolites, namely Ni/Pt-ZSM-5, Ni/Pt/MCM-48, and Ni/Pt-H-beta, have hydrophilic properties which cause them to absorb water when exposed to the atmosphere. The creation of the framework vibration band at 553 cm^−1^ is believed to be due to the dual five-ring structure of MFI-type zeolites. The intensity of the peak at 553 cm^−1^ can be used to determine the level of crystallinity of these zeolites. The XRD and IR experimental data indicate that the MFI-type Ni/Pt-ZSM-5, Ni/Pt/MCM-48, and Ni/Pt–H-beta zeolites have been successfully synthesized.^53,54^

### 3.3. Surface area analysis (BET)

**Fig. 3:**
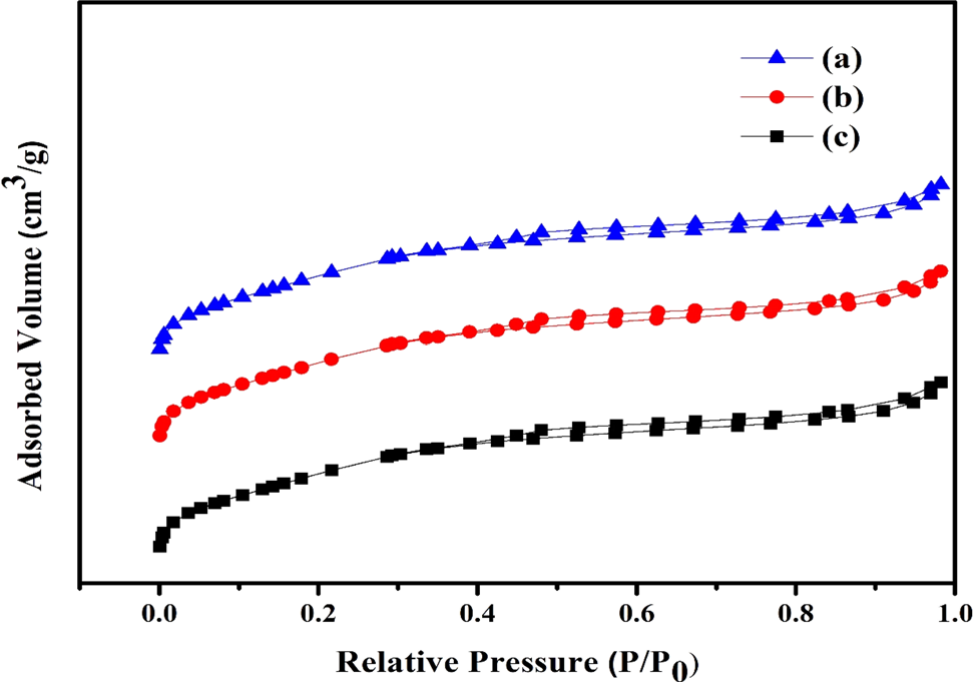
N_2_ sorption studies (a) Ni/Pt-ZSM-5, (b) Ni/Pt-MCM-48, and (c) Ni/Pt-H-beta catalyst.

In Fig. 3 the synthesized Ni/Pt-ZSM-5, Ni/Pt/MCM-48, and Ni/Pt-H-beta zeolite's nitrogen adsorption and desorption isotherms are displayed. A type IV isotherm may be seen in every sample. In this study involving Ni/Pt-ZSM-5, Ni/Pt/MCM-48, and Ni/Pt–H-beta, the formation of more mesopores^55^ resulted in a broader hysteresis loop between *P*/*P*_0_ = 0.45 to *P*/*P*_0_ = 1. Table 1 lists the various zeolite surface areas and pore volumes that were experimentally calculated for Ni/Pt/ZSM-5, Ni/Pt/MCM-48, and Ni/Pt/H-beta. According to Table 1, the BET surface areas and pore volumes of Ni/Pt-ZSM-5, Ni/Pt/MCM-48, and Ni/Pt-H-beta are 394.02 m^2^ g^−1^, 0.458 cm^3^ g^−1^, 368.77 m^2^ g^−1^, 0.392 cm^3^ g^−1^, 360.10 m^2^ g^−1^, and 0.321 cm^3^ g^−1^ respectively, with macrospore volumes of 0.08 cm^3^ g^−1^, 0.09 cm^3^ g^−1^, and 0.34 cm^3^ g^−1^. All three distinct samples all have virtually identical macrospore volumes. These findings show that the extra mesoporosity was produced while the microporosity was preserved. Because Ni/Pt/ZSM-5 has a larger mesoporosity than Ni/Pt/MCM-48 and Ni/Pt–H-beta, reactant molecules diffuse more strongly as a result. The Ni/Pt/ZSM-5 zeolite has a larger surface area and pore volume than the Ni/Pt/H-beta zeolites. The results showed that adding a non-ionic surfactant during the synthesis allowed for the formation of extra mesoporosity in Ni/Pt/MCM-48 samples without compromising the microporosity.^56^

**Table 1:** Surface areas and pore volumes of the catalysts

Catalyst	*S*_BET_ a (m^2^ g^−1^)	Pore volume^b^ (cm^3^ g^−1^)	*D*_p_ c (nm)	Acidity (mmol g^−1^)^2^ total^c^	*LT peak^c^	*HT peak^c^	Si/Al^d^	Ni^d^ (wt%)	Pt^d^ (wt%)
Ni/Pt-ZSM-5	394.02	0.458	4.14	1.56	0.96	0.60	49.2	1.1	0.5
Ni/Pt/MCM-48	368.77	0.392	4.05	1.48	0.92	0.56	49.25	0.98	0.51
Ni/Pt-H-beta	360.10	0.321	3.66	1.40	0.86	0.56	48.70	1.2	0.49

a Measured by the *t*-plot method.

b *V*_meso_ = *V*_total_ − *V*_micro_.

c Total acidity was determined by the standard temperature-programmed desorption of ammonia (TPDA) method. *LT = low temperature, *HT = high temperature.

d Chemical composition of the samples, measured by ICP-OES.

### 3.4. HR-TEM analysis

**Fig. 4:**
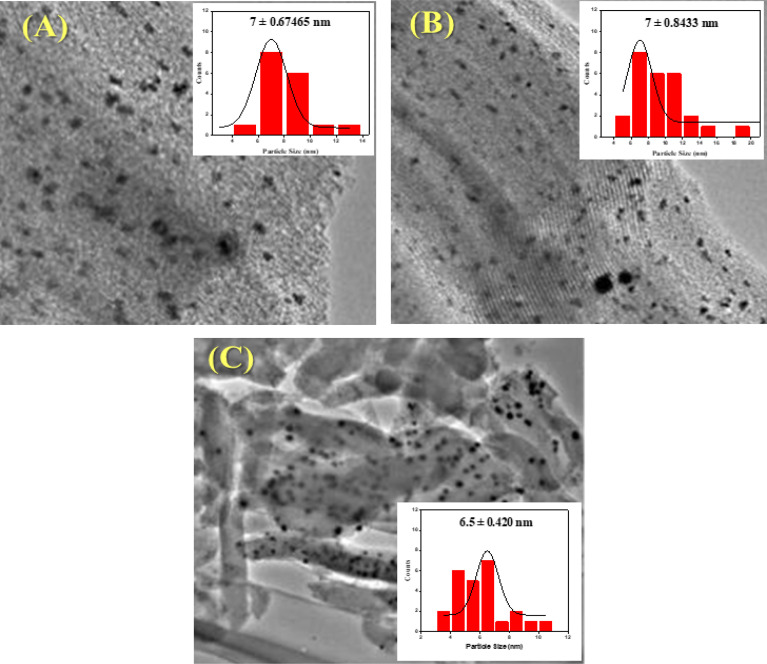
HR-TEM images and particle size distribution shows (A) Ni/Pt-ZSM-5, (B) Ni/Pt-MCM-48, and (C) Ni/Pt-H-beta catalyst.

Transmission electron microscopy was used to determine the morphology and size of the particles of the catalyst. Fig. 4 shows TEM images of Ni/Pt/ZSM-5. Ni/Pt/MCM-48 and Ni/Pt/H-beta and histogram of Ni/Pt nanoparticles distribution. It is clear that Ni/Pt particles (dark and scattered dots in the Fig. 4) are loaded on the carrier channel. Obviously, the shape and size of Pt particles are more uniform on Ni/Pt/ZSM-5, indicating good dispersion of Ni/Pt, while Pt particles on Ni/Pt/ZSM-5 have obvious agglomeration phenomenon.^57^ The average diameter of Pt particles can be calculated by formula (3-1), where, *d*_s_ represents the average particle size nm; *d*_i_ represents the diameter of the Ni/Pt particle, nm; *n*_i_ represents the number of Ni/Pt particles with diameter di. The number of Ni/Pt particles was not less than 200. The calculation results showed that the average particle size of Ni/Pt/ZSM-5 was lower than the value of Ni/Pt/MCM-48 under different sizes of electron microscope images, and Ni/Pt particles on and Ni/Pt/H-beta were more concentrated according to the particle size distribution of Ni/Pt particles, which indicated that the introduction of amino group could effectively disperse Ni/Pt particles on the surface of the carrier.^58^

### 3.5. HR-SEM analysis

**Fig. 5:**
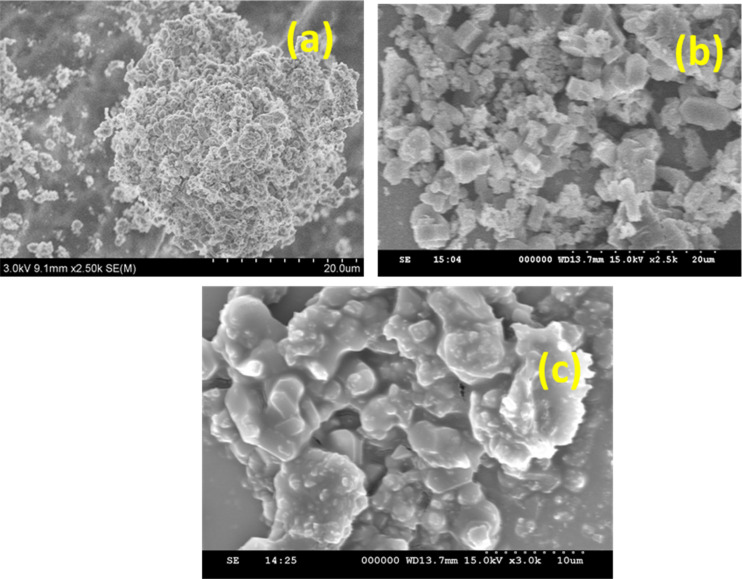
High resolution scanning electron microscopy (HR-SEM) images of (a) Ni/Pt-ZSM-5, (b) Ni/Pt-MCM-48, and (c) Ni/Pt-H-beta catalyst.

The surface morphology analysis of Ni/Pt bimetal supported by ZSM-5, MCM-48, and H-beta catalysts illustrated in Fig. 5 intends to demonstrate distinctions in morphological regularity in shape, and distribution of average crystal size. The observation reveals that the grains are homogeneous in nature. It is apparent that bimetallic loading occurred and the particles are smaller in size and slightly clumped together. The cubic phase MCM-48 sample is composed of sub-micrometre sized aggregated spherical shaped particles, depicted in Fig. 5b. Considering the size of Ni is larger and in the nature of polyatomic anions, apparently to exist as a baffle, prohibiting particles from accrued. The particle size of composite catalysts is not uniform, and they were inter-grown with one another. The bimetals incorporated in the H-beta matrix are obviously apparent as crystalline in Fig. 5c. Fig. 1c depicts sharp and strong crystallinity, among other catalysts. Larger solid shell-like particles were also spotted. It's also interesting to recognize that the morphological image portrays the spheroid-like form and uneven grain shapes. The particle becomes denser, and some particles are agglomerated. Furthermore, it can be indicated, the integration of homogenous distribution Pt and Ni micro-particles influenced the surface shape of the catalyst (Fig. 5a–c). Pt was scattered as small particles ranging in size from 80 to 200 nanometers, whereas Ni was deposited as clusters.

### 3.6. Pyridine – IR analysis

**Fig. 6:**
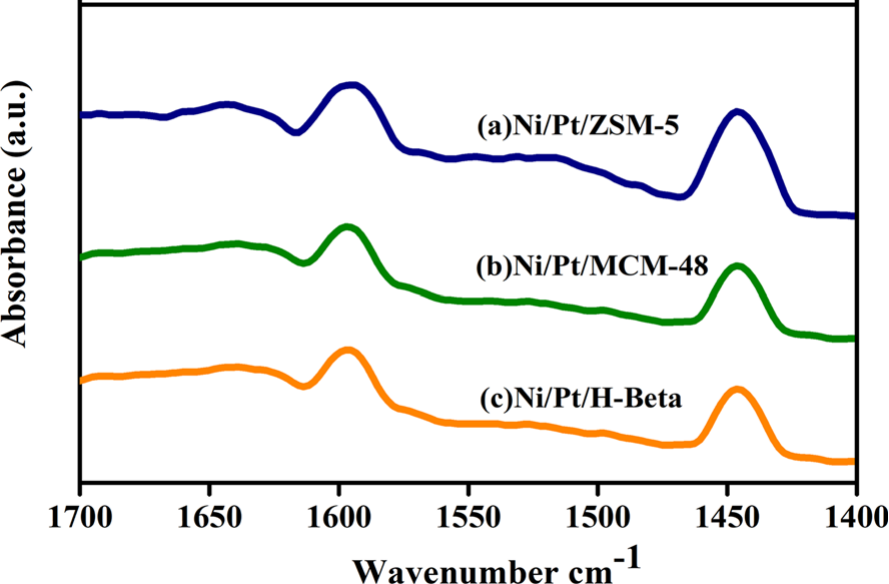
Pyridine FTIR spectra of (a) Ni/Pt-ZSM-5, (b) Ni/Pt-MCM-48, and (c) Ni/Pt-H-beta catalyst.

In this study, Ni/Pt-ZSM-5, Ni/Pt-MCM-48, and Ni/Pt-H-beta were examined for their acidic properties using Py-FTIR. Py-FTIR spectra provide information on the relative changes in the Brønsted and Lewis acid sites. As shown in Fig. 6, the Py-IR spectra reveal that the bands at 1565 and 1445 cm^−1^ are assigned to the pyridine adsorbed on the Brønsted acid sites (BAS) and Lewis acid sites (LAS), respectively.^59^ Relative to Ni/Pt-ZSM-5, the BAS amount slightly decreases in Ni/Pt-MCM-48 and Ni/Pt–H-beta due to the desilication that accompanies the removal of the framework Al species. However, the LAS amount in Ni/Pt-ZSM-5 increases. This indicates that the alkaline treatment's adverse effects on the zeolite's acidity can be effectively weakened when the template is present inside ZSM-5 micropores.^60^ Ni/Pt-MCM-48 has almost the same LAS and BAS amounts as those for Ni/Pt-H-beta, which supports this claim. Ni/Pt-ZSM-5 catalysts had a higher increase in LAS than the Ni/Pt-MCM-48 and Ni/Pt-H-beta catalysts due to the mesoporous structure and high surface area of the Pt-ZSM-5. This increases Ni^2+^ accessibility, creating additional anchoring sites for the Ni^2+^ sites that end up in a highly dispersed state and a low proportion of NiO. The Ni/Pt-ZSM-5 catalyst has the highest proportion of the framework bounded, exhibiting the highest LAS.^61^

### 3.7. NH_3_-TPD analysis

**Fig. 7:**
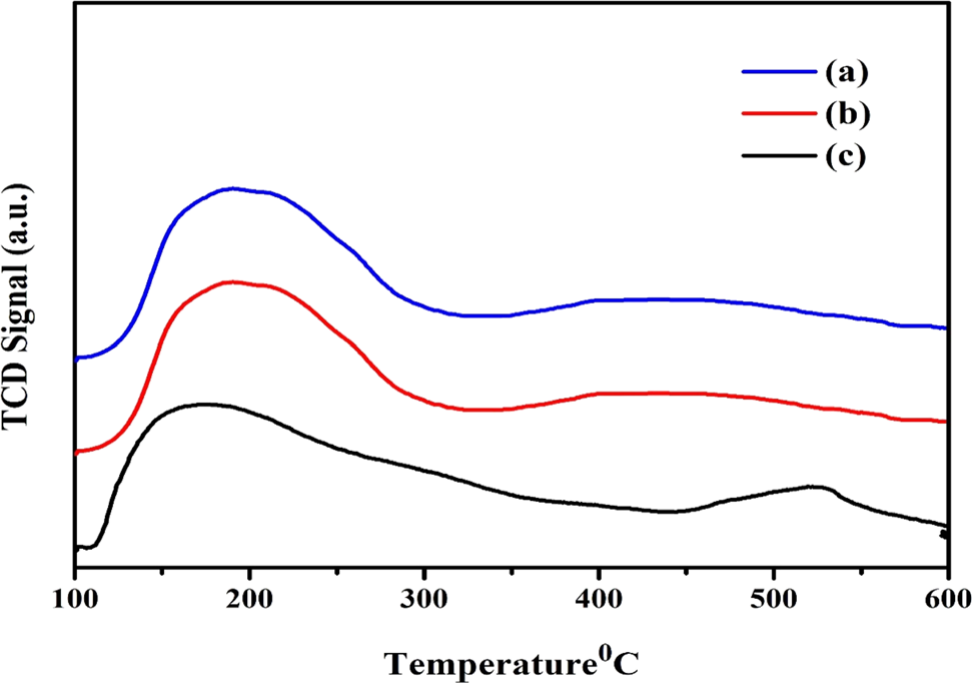
TPD analysis of (a) Ni/Pt-ZSM-5, (b) Ni/Pt-MCM-48, and (c) Ni/Pt-H-beta catalyst.

Using the NH_3_-TPD technique, the acidic properties of the Ni/Pt-ZSM-5, Ni/Pt/MCM-48, and Ni/Pt-H-beta zeolite catalysts are assessed. Fig. 7 shows the corresponding TPD profiles for the Ni/Pt/ZSM-5, Ni/Pt/MCM-48, and Ni/Pt/H-beta zeolites.^62^ Ni/Pt/ZSM-5, Ni/Pt/MCM-48, and Ni/Pt/H-beta zeolite have two desorption peaks that may be seen at roughly 200 °C (low-temperature peak) and 400 °C (high-temperature peak), as shown in the illustration. Although the low-temperature peak is caused by ammonia adsorption from weak acid sites (Brønsted and Lewis acid sites), the high-temperature peak is caused by ammonia desorption from strong acid sites (Brønsted and Lewis acid sites). Zeolites made of Ni/Pt/ZSM-5, Ni/Pt/MCM-48, and Ni/Pt/H-beta have comparable ammonium desorption profiles. In comparison to the Ni/Pt/ZSM-5, Ni/Pt/MCM-48, and Ni/Pt/H-beta zeolite, all zeolite has higher peak intensity.^63^ Due to the tiny amount of Ni/Pt contained in the zeolite structure, the Ni/Pt-H-beta acid strength is weaker. The macrospore walls' amorphous aluminium silicate content is the cause of the Ni/Pt/MCM-48 zeolite's low acid strength. XRD examination supports the amorphous character of the Ni/Pt-ZSM-5, Ni/Pt/MCM-48, and Ni/Pt-H-beta zeolites.

## 4. Catalytic activity study

### 4.1. Effect of supports

**Fig. 8:**
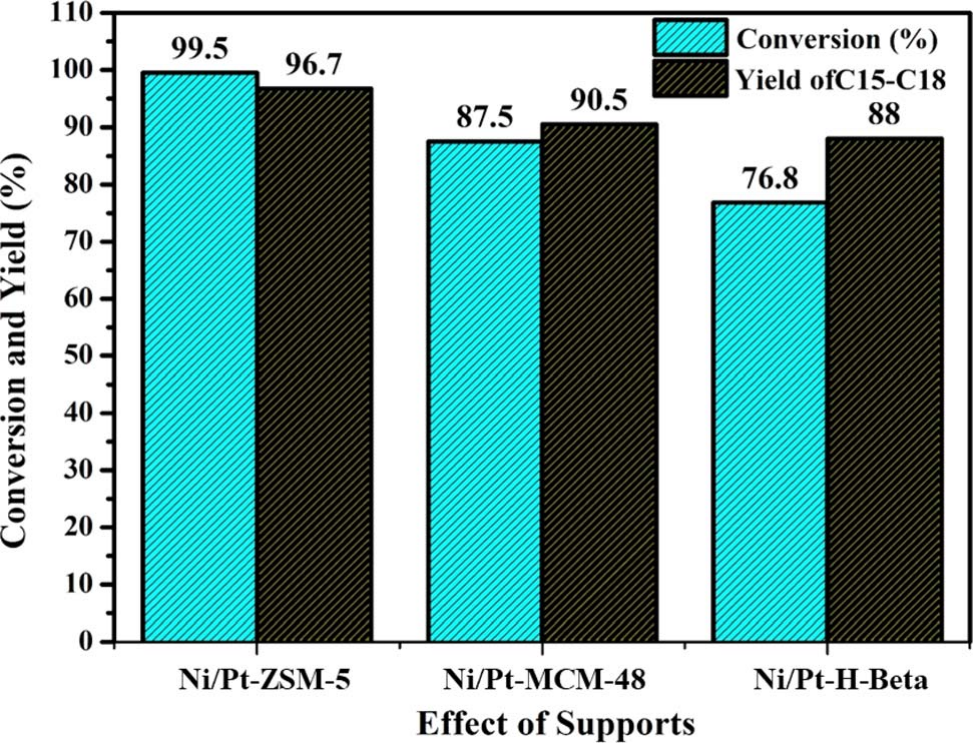
Effect of supports at constant pressure 30 bar and temperature 350 °C reactant: 7% jatropha oil, WHSV = 1 h^−1^, reaction time: 8 h; H_2_ flow rate = 3600 mL h^−1^.

In this study, Ni–Pt catalysts with different aluminium silicate supports, including Ni–Pt/ZSM-5, Ni–Pt/MCM-48, and Ni–Pt/H-beta were synthesized and evaluated for jatropha oil hydrodeoxygenation (HDO) are showed in Fig. 8. The trials were carried out at a stable temperature of 350 °C and a pressure of 30 bar, with all catalysts subjected to the same circumstances.

The catalyst's performance can be attributed to several factors, including the presence of weak acid sites, which were found to be more abundant in Ni/Pt/ZSM-5 based on NH_3_-TPD data (1.60 mmol NH_3_ per g cat.). The catalyst's surface area, pore size, and pore volume also contribute to its catalytic efficiency, with the Ni/Pt/ZSM-5 catalyst demonstrating better performance compared than the other supported catalysts. The Ni/Pt-MCM-48 catalyst had the lowest activity, which could be attributed to a lack of active sites on the surface of the catalyst. The Ni–Pt catalyst supported on ZSM-5 has a better catalytic efficiency than the other supported catalysts. In terms of catalytic activity, the catalysts were found to be most effective in the following order: Ni–Pt/ZSM-5 > Ni–Pt/MCM-48 > Ni–Pt/H-beta.

### 4.2. Molecular weight distribution of jatropha oil

Using gel permeation chromatography (GPC) and polystyrene standards, the molecular weight distribution of jatropha oil was evaluated. The dispersion of Mwt in the jatropha oil was fairly wide. The reaction parameters are 8.8% jatropha oil decalin, 1 h^−1^ WHSV, 9 hours, 50 mL min^−1^ H_2_ flow, 325 °C temperature, and continuous hydrogen pressure of 30 bar. The findings of the GPC analysis revealed that there were initially three different peak types with the retention periods of 9.3, 9.7, and 10.2 min, respectively, with the average corresponding Mwt of 1765, 877, and 457. Following the monitoring of catalytic formation in terms of Mwt, the reaction time (RT) was discovered to be 9.2, 9.5, and 10.3 min, which were changed to 9.7 and 10.8 min with an average matching Mwt 907 and 108. It was determined that this demonstrates unequivocally that, in relation to temperature and pressure, the GPC peaks consistently migrated towards the lower molecular weight region. This could mean that additional depolymerization has taken place, which decreased the intensity. The main long chains were therefore transformed into C_15_–C_18_ hydrocarbons, it can be deduced. With the following average Mwt = 485 and 180, respectively, the retention duration decreased even more when raising the temperature to 350 °C. The conversion of all types of long chains into C_15_–C_18_ hydrocarbons produced the same results. Additionally, the findings of the GC analysis showed the same kind of results. The retention times were 9.7 and the 10.2nd section min with average Mwt = 390 and 180, respectively, despite raising the temperature to 375 °C. However, because of the increased quantity of cracking and the deposits that covered the surface of the sites that were active, C_1_–C_14_ hydrocarbons were produced, which led to the development of coke. Therefore, as demonstrated it may be caused by the blocking of acidic sites on the outermost layer brought on by the compounds' splitting into lower hydrocarbon. According to the GPC findings, 350 °C was the ideal temperature whereby more conversion and more specifically (C15–C18 hydrocarbon) were seen.^64^

### 4.3. Effect of temperature

**Fig. 9:**
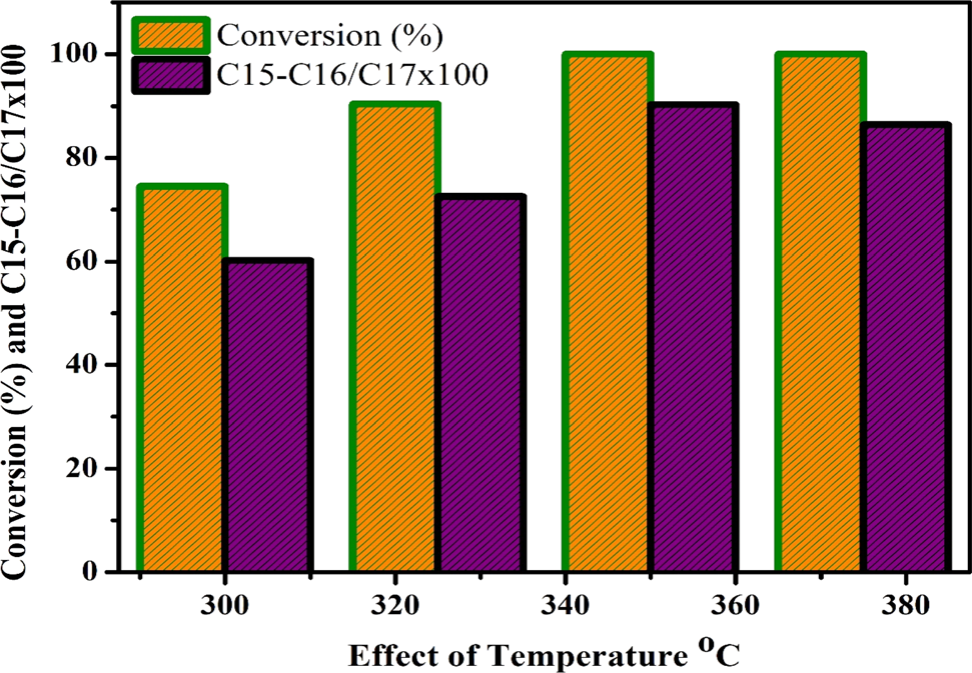
Effect of temperature, HDO of jatropha oil for conversion and C15–C16/C17–C18 ratio at constant pressure 30 bar, WHSV = 1 h^−1^, reaction time: 8 h; H_2_ flow rate = 3600 mL h^−1^.

Fig. 9 depicts the effect of temperature on the hydrodeoxygenation (HDO) of jatropha oil employing a Ni/Pt catalyst supported on mesoporous aluminium silicate Ni/Pt-ZSM-5. The process involved converting the oil into biofuel under continuous H_2_ pressure of 30 bar (435 psi) and temperatures ranging from 300 to 375 °C. The conversion of liquid products reached 74.5% and 87.5% at 300 °C and 325 °C, respectively has a preference for C_15_, C_16_, C_17_, and C_18_ carbon chain compounds. The selectivity percentages for these products were 23.5%, 31.48%, 15.32%, and 23.45% at 300 °C, and 18.5%, 27.3%, 16.5%, and 26.4% at 325 °C. Additionally, cracking products of C_5_–C_14_ (3% and 5%) and lower carbon compounds of C1–C4 (6.5% and 7.6%) were also obtained. As the temperature was raised to 350 °C, the conversion reached 100%, and higher selectivity was observed for hydrocarbons with C_15_, C_16_, C_17_, and C_18_ carbon chains, with respective formation rates of 13.66%, 26.10%, 20.2%, and 28.6%. The presence of by-products, such as lower alkanes (C_1_–C_4_ at 4–6%) and cracking products (C_5_–C_14_ at 8.4%), C_15_, C_16_, C_17_, and C_18_ conversion and selectivity were greatly improved. However, with a further increase in temperature to 375 °C, the conversion slightly decreased to 98.2%, and selectivity for alkanes with carbon chains of C_15_, C_16_, C_17_, and C_18_ was discovered (13%, 26.5%, 22.5%, and 27.6%). At this higher temperature, lower alkanes (C_1_–C_4_ at 3%) were generated as by-products at lower concentrations. Table 2 indicates that at higher temperatures, a greater number of cracking products, including C_5_–C_14_ and >C_4_, were formed due to carbon deposition or coking on the catalyst, which led to the blocking of acidic sites. The HDO of jatropha seed oil revealed that the C_15_–C_16_/C_17_–C_18_ ratio increased significantly in the Celsius range of 300 to 350 °C, with up to 80% selectivity towards the C_15_–C_16_/C_17_–C_18_ ratio after reaching 375 °C. However, this increase in selectivity was accompanied by a decrease in the C_15_–C_16_–C_17_–C_18_ ratio, potentially due to coking or carbon deposition on the catalyst. Based on the data presented in Table 2, the best reaction conditions were found to be 350 °C and 30 bar pressure.^65^

**Table 2:** Effect of temperature, HDO of jatropha oil for C15–C16/C17–C18 selectivity at constant pressure 30 bar, WHSV = 1 h^−1^, reaction time: 8 h; H_2_ flow rate = 3600 mL h^−1^

S. No.	Temperature 2(°C)	<C4	<C14	C15	C16	C17	C18
1	300	6.5	11	24.4	30.3	14.1	20.2
2	325	6.4	10.7	24.5	27.5	14.9	22.5
3	350	4.8	10	17.9	26.8	15.5	29.8
4	375	3.1	13.5	12.7	28.2	21.1	24.6

### 4.4. Effect of WHSV

The influence of weight hourly space velocity (WHSV) on the hydrodeoxygenation (HDO) of jatropha oil using a Ni/Pt/ZSM-5 catalyst was investigated under constant conditions of 350 °C temperature and 30 bar pressure. The WHSV range of 0.5–2.0 h^−1^ was examined to understand its effect on the process. It was observed that as the space velocity increased, the selectivity decreased to an optimal level. Consequently, the conversion decreased from 100% (at 0.5 h^−1^) to 65% (at 2.0 h^−1^). Selectivity for C_14_, C_15_, C_16_, C_17_, and C18 carbon chains fell from 21.5%, 25.5%, 17.5%, and 31.5%, respectively, to 23.5%, 28.5%, 19.5%, and 16.5%. The highest conversion and selectivity were achieved assuming a WHSV of 1.0 h^−1^. However, increasing the WHSV to 2.0 h^−1^ reduced the selectivity of C_14_, C_15_, C_16_, C_17_, and C_18_ carbon chains due to the absence of surface molecule interactions. In particular, the selectivity for C_17_ and C_18_ fell dramatically, resulting in a larger selectivity for C_15_ and C_16_ alone. Table 3 depicts the active regions on the catalyst surface that alter selectivity.

**Table 3:** Effect of WHSV at constant Pressure 30 bar, Reactant: 7% jatropha oil, WHSV = 1 h^−1^, Reaction time: 5 h; H_2_ flow rate = 3600 mL h^−1^

S. No.	Effect of WHSV h^−1^	<C4	<C14	C15	C16	C17	C18
1	0.5	2.6	5.2	21.5	25.7	17.4	30.2
2	1.0	3.2	8	22.5	24.5	16.5	28.5
3	1.5	5.6	14.2	22.2	24.8	18.4	20.4
4	2.0	8.6	20.2	24.8	24.2	16.4	14.4

### 4.5. Time on stream (TOS)

**Fig. 10:**
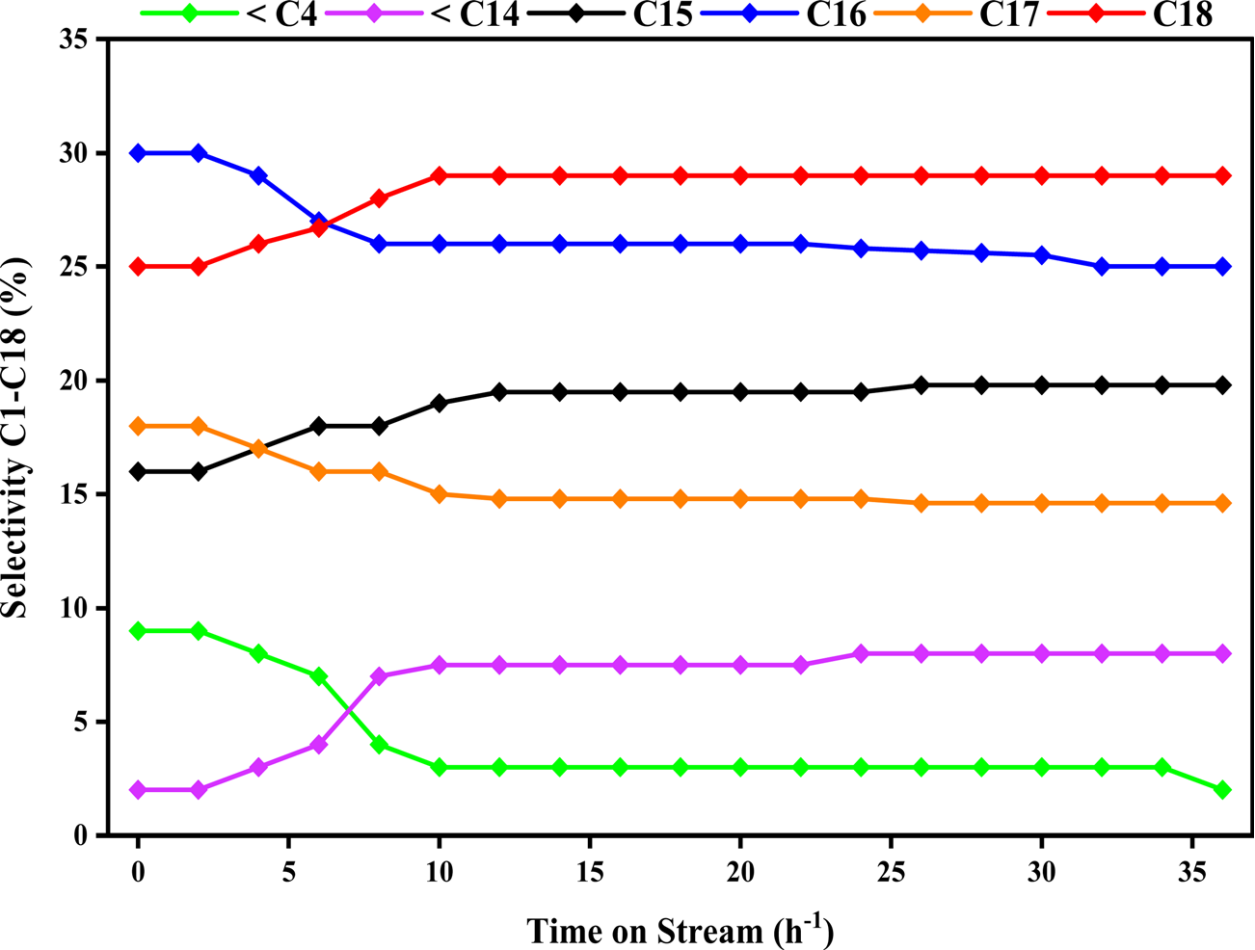
Time on stream at constant pressure 30 bar, temperature 350 °C, reactant: 7% jatropha oil, WHSV = 1.0 h^−1^, reaction time: 8 h; H_2_ flow rate = 3600 mL h^−1^.

Fig. 10 illustrates the selectivity of the Ni/Pt-ZSM-5 catalyst in the conversion of jatropha oil over a period of 36 hours, highlighting the impact of time on stream. Initially, within the first hour, the selectivity of jatropha oil over the Ni/Pt-ZSM-5 catalyst was approximately 68%. The selectivity for C_1_–C_5_, C_6_–C_13_, C_14_, C_15_, C_16_, C_17_, and C_18_ carbon chains was 8%, 3%, 17%, 31%, 17%, and 24% respectively. The resulting products included hydrocarbons ranging from C_1_ to C_18_. The selectivity output of the catalyst increased with time and peaked at about 10 hours. However, the selectivity for C_1_–C_5_, C_6_–C_13_, C_14_, C_15_, C_16_, C_17_, and C_18_ changed to 1.4%, 7.2%, 12.8%, 23.6%, 21.2%, and 28.8% respectively. After the 10th hour, the conversion and selectivity showed minimal changes with time-on-stream and reached a steady state.

### 4.6. Stability of used catalyst

**Fig. 11:**
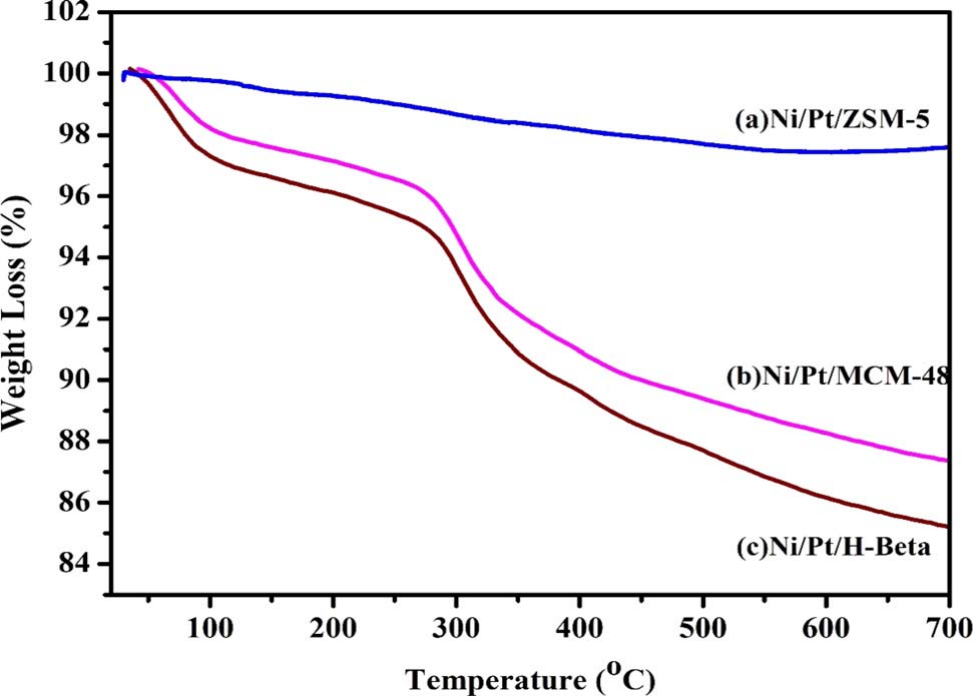
TGA analysis for used catalyst (a) Ni/Pt-ZSM-5, (b) Ni/Pt-MCM-48, and (c) Ni/Pt-H-beta catalyst.

Fig. 11 displays the TGA profiles of the spent catalyst used in the HDO reaction. However, in the case of acidic supports, particle sintering and coke formation occur during the reaction. Ni/Pt-MCM-48 and Ni/Pt-H-beta showed a significant amount of weight loss and at temperature around 300 °C the reduction in weight as about 12%. However, the reduction in weight for Ni/Pt-ZSM-5 was about 3%, indicating that the higher efficiency of conversion has resulted in lower carbon fouling of the catalyst. During the stability test, the material displayed a constant catalytic behavior, which is consistent with the highly hydrogenated coke that can be observed. This results in most of the reactive sites remaining accessible for reaction. This is a positive outcome as it indicates that bi-metallic Ni and Pt catalysts on ZSM-5 are capable of easy regeneration compared with other catalysts.

### 4.7. Proposed mechanism of non-edible oil

**Fig. 12:**
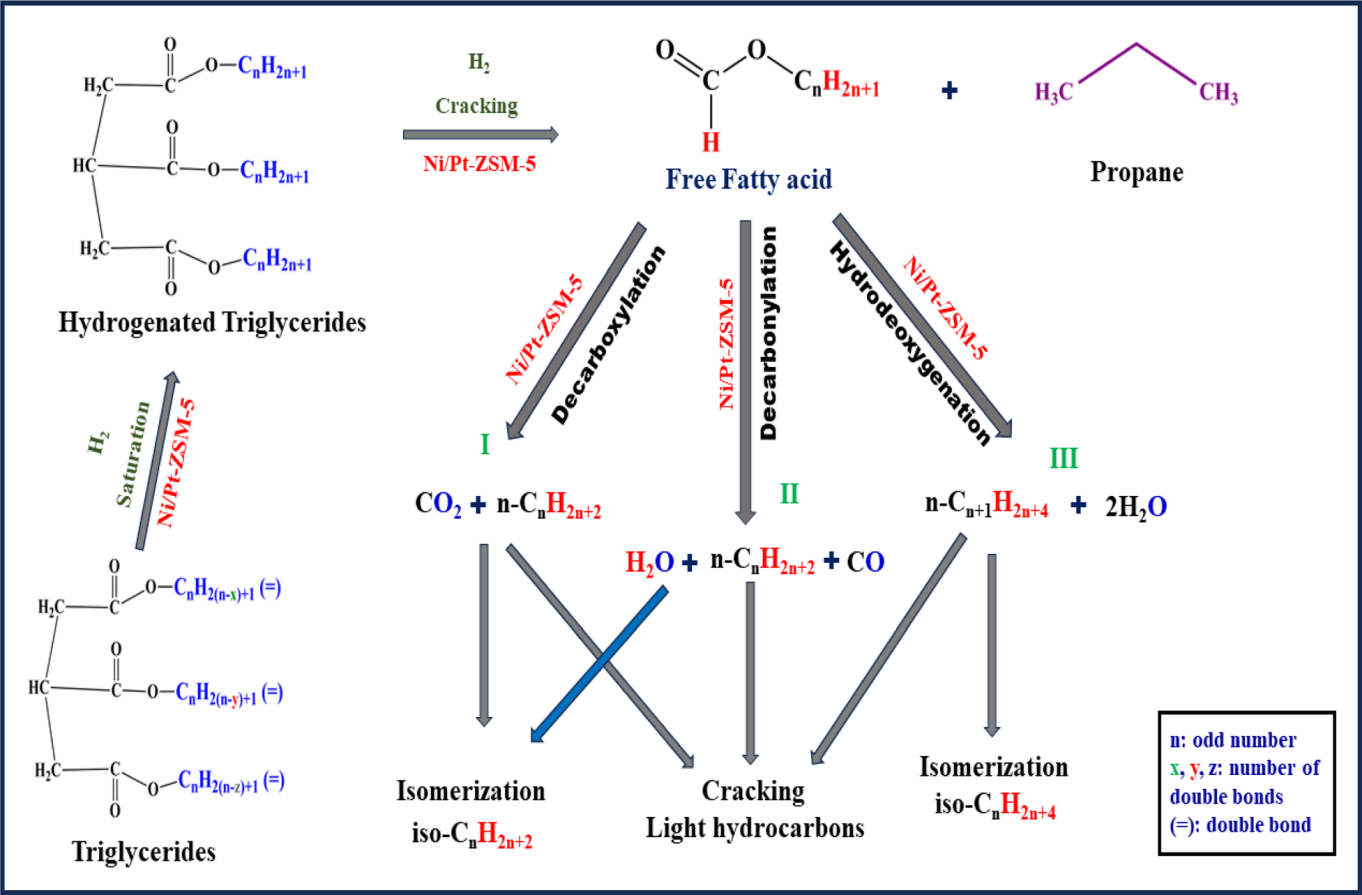
Proposed mechanism for HDO of jatropha oil over Ni–Pt/supported catalysts.

The integration of Ni–Pt bimetals onto various supports was studied for triglyceride hydrodeoxygenation (HDO). Fig. 12 presents one of the pathways observed during the reaction. The fundamental goal of the HDO procedure is to decrease the O/C ratio while increasing the H/C ratio. The triglyceride passes through various transformations, including hydrodeoxygenation, decarbonylation, and decarboxylation, which results in hydrocarbon conversion. The hydrodeoxygenation reaction is a process that involves the removal of oxygen in the presence of hydrogen. Oxygen atoms usually contain unpaired electrons that are more basic than the pi-electrons of long-chain hydrocarbons. As a result, the oxygen atoms connected with C=O and C–OH groups (long-chain hydrocarbons) are absorbed on the Brønsted acid sites of the catalysts. Subsequently, C–O bond cleavage takes place on the catalyst surface and hydrogen active sites.

Initially, the triglyceride is hydrogenated or saturated. These active acid sites are critical in the generation of propane/propanol and the subsequent cracking products. The following phase involves the conversion of long-chain fatty acids that are free into hydrocarbons with lengthy chains and isomers. The oxygen removal process involves the selective production of hydrocarbons in the presence of Ni–Pt including the hydrogenation of C=O bonds and the cleavage of C–O and C–C bonds. C–O bond cleavage can occur by dehydrogenation followed by direct cleavage catalyzed by metallic sites or *via* dehydration catalyzed by acidic sites linked with Ni– Pt/supports. By-products such as water, carbon dioxide, and carbon monoxide are generated during the reaction. During depolymerization, fatty acids are formed, followed by isomerization products. In the first route, decarboxylation takes place, followed by dehydration through *E*_2_ elimination, resulting in the production of long-chain alkenes. In the second route, decarboxylation is followed by the conversion of alkene to alkane in the presence of a catalyst. In the third route hydrodeoxygenation of jatropha oil, the main products are typically hydrocarbons with reduced oxygen content. These liquid products were also composed of C15–C18 straight-chain alkanes, a few iso alkanes, aromatics, and naphthene on Ni/Pt-ZSM-5 bimetallic catalysts. The mechanism depicted in Fig. 12 demonstrates how catalyst acidity, pressure, and temperature influence isomer production. These compounds exhibit improved characteristics, such as higher cetane numbers, compared to traditional petroleum-based fuels.

## 5. Conclusion

For the hydrodeoxygenation (HDO) of non-edible oils, a variety of Ni/Pt catalysts based on mesoporous materials which include ZSM-5, MCM-48, and H-beta were synthesized and tested. Jatropha oil was hydrodeoxygenated in vapour phase in a fixed bed reactor. Various performance indicators, including SRR, turnover frequency (TOF), conversion, stability, and selectivity, were used to analyze the catalytic action of various catalysts. The catalytic activity of the HDO reaction was significantly influenced by modifications in the supports, leading to improved textural properties. Among the catalysts tested, the ZSM-5/Ni/Pt catalyst exhibited superior activity. The enhanced catalytic efficiency was associated with the presence of advantageous physical and chemical features such weak acidic sites, a high surface area for hydrogen consumption, and effective dispersion of active metals. Notably, the ZSM-5/Ni/Pt catalyst outperformed the other supporting catalysts in terms of catalytic activity during the hydrodeoxygenation of jatropha oil. It has been concluded that the ZSM-5/Ni/Pt catalysts had the highest conversion (100%) and selectivity of C_1_–C_5_, C_6_–C_13_, C_14_, C_15_, C_16_, C_17_, and C_18_ were at 7.2, 4.8, 17.2, 22.5, 16.2, and 28.6, respectively. Similarly, the effects of temperature, pressure, WHSV, and time on the stream were examined for the Ni/Pt-ZSM-5 catalyst.

## Conflicts of interest

There are no conflicts to declare.

## Supplementary Material
